# Changes in Ocular Biomechanics During Adolescence and Its Relationship with Lifestyle and Myopic Progression: The Oporto Myopia Study

**DOI:** 10.3390/bioengineering13030367

**Published:** 2026-03-20

**Authors:** Pedro M. L. Baptista, Gabriel Santos, João H. Marques, André Ferreira, Beatriz Vieira, Paulo Sousa, Ricardo Parreira, Renato Ambrósio, Pedro M. A. M. Menéres, João N. M. Beirão

**Affiliations:** 1 Ophthalmology Department, Centro Hospitalar Universitário do Porto, 4099-001 Porto, Portugal; 2 Instituto de Ciências Biomédicas Abel Salazar, Universidade do Porto, 4050-313 Porto, Portugal; 3 Faculdade de Medicina da Universidade do Porto, Universidade do Porto, 4200-319 Porto, Portugal; 4 Rio de Janeiro Corneal Tomography and Biomechanics Study Group, Rio de Janeiro 20520-050, Brazil; 5 Department of Cornea and Refractive Surgery, Instituto de Olhos Renato Ambrósio, Rio de Janeiro 20520-050, Brazil; 6 Department of Opthalmology, Federal University of the State of Rio de Janeiro (UNIRIO), Rio de Janeiro 21941-617, Brazil; 7 Department of Ophthalmology, Federal University of São Paulo (UNIFESP), São Paulo 04039-060, Brazil; 8 Brazilian Study Group of Artificial Intelligence and Corneal Analysis—BrAIN, Rio de Janeiro 20520-050, Brazil

**Keywords:** axial length, corneal biomechanics, Corvis, emmetropization, lifestyle, myopia, myopization, Scheimpflug camera, sum of segments, vitreous cavity

## Abstract

The relationship between lifestyle, ocular biomechanical behavior, and myopia is not well established in the literature. The present study aims to describe changes in ocular biomechanics during adolescence and to explore their relationship with lifestyle factors and myopic progression. Prospective cohort study including 63 adolescents (126 eyes) with a mean age of 14.1 ± 2.6 years old examined twice over a 30 ± 0.9-month period. The data from biomechanics, biometry, corneal tomography, and lifestyle was addressed. The relationships between biomechanical changes, biometric and refractive variation, and lifestyle variables were analyzed using parametric and non-parametric statistics with a significance level of *p* < 0.05. A biomechanical stiffening trend was found. Axial elongation was 0.12 ± 0.17 mm, and refractive shift was −0.32 ± 0.87 D. The history of allergies was associated with greater axial growth (*p* = 0.032) and smaller increase in stress–strain-index (SSI) (*p* = 0.01). Myopization was higher in eyes with ocular surface symptoms (*p* = 0.049) and those with reported eye-rubbing habits (*p* = 0.04), with a lower gain in stiffness (*p* < 0.05). Outdoor activities were associated with higher gain in corneo-scleral stiffness (*p* < 0.05). Reduced myopization correlated directly with the increase in the SSI (*p* < 0.05) and inversely with the Integrated Radius (*p* < 0.05). Greater increases in axial length (AL), vitreous cavity length (VCL), and the ratio between VCL and AL (R_VCL/AL) correlated negatively with the increase in the SSI (*p* < 0.05). The increase in the R_VCL/AL correlated positively with the time spent on digital devices and negatively with the amount of outdoor activity (*p* < 0.05). Biomechanics may represent the physiological bridge between the environmental exposure and myopization, as lower gain in corneo-scleral stiffness was consistently associated with greater axial elongation and refractive myopization, with outdoor activity appearing to be protective.

## 1. Introduction

Myopia is a prevalent and complex ophthalmological condition, estimated to have affected approximately 2.5 billion people globally by 2020 [1,2]. With its prevalence on the rise, projections suggest that by 2050, myopia and high myopia will affect about 50% and 10% of the world’s population, respectively [2]. This increasing trend is expected to have significant economic implications, as robust modeling data from meta-analyses estimated that, in 2015, the global potential productivity loss due to vision impairment amounted to $244 billion from uncorrected myopia and $6 billion from myopic macular degeneration [3].

For decades, the ophthalmology community has been investigating the potential causes behind the rising prevalence of myopia in an effort to mitigate its associated burden. While genetic factors play a significant role, recent decades have shown that in economically developed societies, most cases of myopia emerge during the school years—commonly referred to as school myopia [4]—and only a small proportion of myopia cases are clearly familial, typically characterized by early onset, high severity, defined chromosomal loci, and, in some cases, causal genetic mutations [5]. The increase in myopia parallels the rise in levels of education [6] and decreased time spent outdoors [7,8,9,10].

Despite growing evidence linking children’s lifestyle factors to school-age myopia—particularly highlighted during the COVID-19 pandemic [11]—the biological mechanisms driving myopia progression remain incompletely understood. While the emmetropization theory provides the prevailing conceptual framework, the pathways translating environmental visual stimuli into structural ocular changes are not fully clarified [12,13].

Axial elongation, the structural hallmark of myopia progression, is primarily driven by the remodeling of the posterior sclera. Alterations in scleral biomechanics arise from changes in the extracellular matrix, including collagen fiber organization, cross-linking, and proteoglycan composition [14]. These processes are regulated by molecular signaling pathways involving transforming growth factor-β (TGF-β), matrix metalloproteinases (MMPs), tissue inhibitors of metalloproteinases (TIMPs), and mechanotransduction mechanisms that modulate scleral fibroblast activity [15]. A shift toward a more compliant scleral structure reduces resistance to intraocular pressure-related mechanical stress, thereby facilitating ocular elongation [16].

If environmental factors influence retinal signaling and downstream extracellular matrix remodeling, they may indirectly modulate scleral biomechanics and axial growth. Therefore, investigating ocular biomechanical behavior during childhood and adolescence may provide important insight into the relationship between lifestyle factors and myopia progression.

Nowadays, in vivo data from ocular biomechanical behavior can be obtained through the analysis of corneo-scleral tissue movement using ultra-high-speed Scheimpflug imaging during non-contact tonometry with the Corvis ST^®^ (OCULUS, Wetzlar, Germany) [17] and includes evolving several validated algorithms as descriptors of ocular biomechanical behavior and its stiffness [18,19,20,21,22]. However, the assessment of changes on ocular biomechanics throughout adolescence and its relationship with overall or segmental axial eye growth or with refractive changes have never been explored. Additionally, there is no evidence regarding the possible relationship between lifestyle and these changes during the growth phase in which school myopia develops.

The present study aims to describe the changes in ocular biomechanics during adolescence in a European cohort and to relate it with lifestyle parameters and myopic progression.

## 2. Materials and Methods

### 2.1. Design

This is a prospective cohort study. Data was collected from 2 timepoints, spaced by 30 ± 0.9 months (2.5 years).

The study was conducted in accordance with the Declaration of Helsinki and approved by the Institutional Review Board (IRB) of Centro Hospitalar e Universitário de Santo António (protocol code 2020-201(158-DEFI-160-CE), 13 October 2021).

### 2.2. Setting

Centro Hospitalar e Universitário de Santo António, Oporto, Portugal.

### 2.3. Population Inclusion and Exclusion Criteria

The study population included individuals between 10 and 15 years old consecutively selected from the general ophthalmology clinic at our center. The exclusion criteria were: familial or personal ocular pathologic history besides myopia; amblyopia history: ocular surgery history; ocular inflammation at the time of examinations; corneal leucomas or other corneal or conjunctival pathologies; the inability to perform the exams; and the utilization of myopia control interventions, including atropine, contact lenses, or spectacle lenses with specialized optical designs.

### 2.4. Ocular Biomechanical Data

Biomechanical assessment was made at both timepoints by means of Scheimpflug camera, with Corvis ST^®^ (OCULUS, Wetzlar, Germany), through the dynamic corneal response (DCR) parameters. Only exams with ‘OK’ quality score were included. Both first-generation parameters—derived from the direct analysis of the corneal excursion image in different timepoints, including the time of first and second applanations (A1 and A2), the time of the highest corneal concavity (HC), and maximum whole eye movement (WEM)—and second-generation parameters, such as algorithms including biomechanically corrected-IOP 1 (bIOP), were considered. Corvis biomechanical index (CBI), tomographic and biomechanical index (TBI), stiffness parameter in A1 (SP-A1) and in HC (SP-HC), and stress–strain index (SSI) were analyzed. Deltas of variation were built for all parameters. Table 1 summarizes all Scheimpflug-based parameters used in the study and their explanation, including which tissue each of them is most associated with and the theoretically expected behavior in the presence of higher values.

### 2.5. Ocular Biometric Data

Biometric data from axial length (AL) and segmental ocular biometric lengths, including central corneal thickness (CCT), anterior chamber depth (ACD), and lens thickness (LT), were assessed at both timepoints by means of swept source optical coherence tomography (OCT) with the IOL MASTER 700^®^ (ZEISS, Oberkochen, Germany). The variable vitreous cavity length (VCL) was built [VCL = AL − (CCT + ACD + LT)]. The ratio between VCL and AL (R_VCL/AL) was calculated, and deltas of variation were built for AL (Δ_AL), VCL (Δ_VCL), and R_VCL/AL (Δ_R_VCL/AL).

### 2.6. Objective Refraction Data

Non-cycloplegic objective refractive status—(sphere (S), cylinder (C), and spherical equivalent (SE))—was assessed at both timepoints through the KR-800 Auto Kerato-Refractometer^®^ (TOPCON, Livermore, California (CA), United States of América). Deltas of variation were built for S, C, and SE (Δ_S, Δ_C, and Δ_SE).

### 2.7. Corneal Tomography Data

Keratometric values—minimum (K1) and maximum (K2)—were assessed through Pentacam HR^®^ (OCULUS, Wetzlar, Germany) at both timepoints, and deltas of variation were built (Δ_K1 and Δ_K2).

### 2.8. Lifestyle Data

General health data and lifestyle data from the follow-up interval were collected at the end of the follow-up through a questionnaire accepted and validated by the Centro Hospitalar e Universitário de Santo António Ethical Commission (nr 158-DEFI/160-CE), including age and questions regarding familiar history of myopia, allergies, sleeping position, dry eye symptoms, eye-rubbing habits, sun exposure, physical activity, digital device utilization, and reading habits. The anonymized data was collected via *Google Form*^®^ (Supplementary Materials—Questionnaire Oporto Myopia Study). Filling out the form was done by the individual with the help of parents when necessary.

### 2.9. Step 1—Inter-Individual Analysis

The variation (Δ) in the biomechanical parameters and ocular biometric, tomographic, and objective refraction data were compared to address the differences between individuals with family history of myopia, those with reported allergic diseases, and those with predominantly ventral sleeping position.

### 2.10. Step 2—Inter-Eye Analysis

The variation (Δ) in the biomechanical parameters and ocular biometric, tomographic, and objective refraction data were compared to address the differences between the groups of eyes with predominantly ipsilateral sleeping position, those with dry eye symptoms, and those with eye-rubbing habits but without these conditions.

### 2.11. Step 3—Study of Correlations

The variation (Δ) of ocular biomechanical parameters was correlated with the variation (Δ) in ocular biometric and objective refraction parameters and with the individual lifestyle variables of weekly hours of outdoor activity, weekly hours of physical activity, daily hours spent on digital devices, and daily hours spent reading/writing.

### 2.12. Statistical Analysis

Descriptive statistics of the dataset were calculated for ocular biomechanical, biometric, and refractive data. Normality of the data was tested with the Shapiro–Wilk and Kolmogorov–Smirnov tests. When the parametric analysis could be applied, the Student *t*-test was used to compare the variables. When non-parametric tests were needed, the Mann–Whitney test was applied. Pearson correlation coefficient and Spearman rank correlations were used in the correlation analysis. All analyses were performed using SPSS v26.0. All values are shown as mean ± standard deviation unless otherwise specified. All *p*-values (*p*) were two-sided, and *p*-values of < 0.05 were considered significant.

## 3. Results

The present study analyzed a prospective cohort including 63 adolescents (126 eyes) with a mean baseline age of 14.1 ± 2.6 years old.

Regarding baseline biometry, approximately 61% of eyes had a baseline axial length (AL) between 23 and 25 mm, while 13.5% had an AL greater than 25 mm. Concerning baseline refractive status, there was an equal distribution among eyes with myopia greater than 1 D, eyes with myopia less than 1 D, and hyperopic eyes. During follow-up, the mean ocular growth (ΔAL) was 0.123 ± 0.17 mm, and the mean myopic shift (ΔSE) was −0.321 ± 0.87 D. Biomechanical assessment revealed an overall increase in ocular stiffness over time, with mean increases in SSI, SP-A1, and SP-HC of 0.040 ± 0.12, 2.461 ± 13.2, and 0.422 ± 2.6, respectively. Additionally, a mean decrease of −0.394 ± 0.86 was observed in the integrated radius parameter.

Approximately 38% had a family history of myopia in at least one parent, 43% reported a history of allergies, and around 60% indicated the ventral position as their predominant sleeping posture. The data for each eye showed that about 41% of eyes were habitually subjected to an ipsilateral sleeping position, and symptoms of ocular surface discomfort and eye-rubbing habits were reported in approximately 44% and 67% of eyes, respectively. On average, outdoor activities occupied 3.4 ± 1.9 weekly hours and digital devices were used during 3.3 ± 1.5 daily hours.

In individuals with a family history of myopia, a significant decrease in A2 time (−0.014 ± 0.07 vs. 0.037 ± 0.06; *p* = 0.045) was observed during the follow-up, and the integrated radius function showed a significantly greater reduction (−0.644 ± 0.04 vs. −0.232 ± 0.068; *p* = 0.006) (Table 2). Individuals with allergies exhibited significantly greater axial growth (0.155 ± 0.2 vs. 0.087 ± 0.09; *p* = 0.032) and a significantly smaller increase in the SSI (0.012 ± 0.1 vs. 0.070 ± 0.13; *p* = 0.010). Individuals with a predominantly ventral sleeping position showed an increase in A1 velocity (0.004 ± 0.02 vs. −0.002 ± 0.01, *p* = 0.046) and a greater increase in HC DArc length (0.013 ± 0.2 vs. 0.003 ± 0.02, *p* = 0.049) (Table 2).

The eyes with ocular surface symptoms exhibited a higher degree of myopization (−0.49 ± 0.8 vs. −0.17 ± 0.9; *p* = 0.05), an increase in A1 deflection length (0.037 ± 0.31 vs. −0.019 ± 0.15; *p* = 0.032) and A1 deflection amp (0.002 ± 0.01 vs. −0.018 ± 0.02; *p* = 0.031) and an increase in the TBI (0.057 ± 0.17 vs. −0.040 ± 0.15; *p* < 0.001). The eyes with eye-rubbing habits showed a higher degree of myopization (−0.41 ± 0.79 vs. −0.125 ± 1.0; *p* = 0.04), a smaller increase in radius (0.193 ± 0.69 vs. 0.432 ± 0.46: *p* = 0.018), and an increase in A2 arc length (0.007 ± 0.03 vs. −0.001 ± 0.008; *p* = 0.032) (Table 3).

Weekly hours of outdoor activity, weekly hours of physical activity, daily hours spent on digital devices, and daily hours spent reading/writing were correlated with the variation in several biomechanical parameters during the follow-up period (*p* < 0.05) (Table 4).

We found correlations between the variation in several biomechanical parameters and Δ_SE, Δ_AL, Δ_VCL, and Δ_R_VCL/AL during the follow-up period (*p* < 0.05) (Table 5).

## 4. Discussion

The present study aimed to examine whether ocular biomechanics could play a pivotal role in the relationship between the myopization process and individuals’ lifestyles in a cohort of European Caucasian adolescents. Additionally, it describes the changes in ocular biomechanics during adolescence and investigates the relationship between these changes and variables related to family history and behavioral factors.

To the authors’ knowledge, this is the first study to describe the variation in ocular biomechanic behavior during adolescence in a European population. Thus, it may represent an important contribution toward establishing a normative range for these changes in biomechanics, which could eventually become an integral part of the myopization process assessment in the future.

This sample included predominantly eyes within the European normative AL range [23] and with low refractive error from individuals with a mean baseline age of 14 years old, which enhances the relevance of the results in the context of school myopia appearing in otherwise normal eyes. Additionally, regarding the inter-individual and inter-eye analysis, the n in each group was homogeneous, allowing for more consistent conclusions from the results.

Through the lifestyle-related analysis, the present study demonstrates a concerningly high ratio between the high amount of time that adolescents spend using digital devices and the low amount of time that they spend on outdoor activities, and this may explain the founded high prevalence of ocular surface symptoms and eye-rubbing habits.

Since the evidence suggest that the emmetropization process may involve the scleral tissue as a key substrate [24,25,26], potentially mediated through dopamine pathways [27] under direct genetic influence [28], we consider ocular biomechanics as pivotal in understanding the underlying mechanisms. Its assessment has made significant advancements since the ORA [29] to evolve with the inclusion of a Scheimpflug camera [17], which enabled the visualization of corneal excursion and the accessory lateral movement of the corneo-scleral transition. As a result, biomechanical analysis is no longer binary—classifying the cornea as simply more or less rigid—but rather the description of a unique biomechanical fingerprint for each individual. Although a detailed explanation of each parameter is beyond the scope of this study, it is important to highlight some that can be important in the myopization setting: (1) IR is an important descriptor of total cumulative corneal deflection, representing the area under the curve of the movement function until it returns to its initial position [17]; (2) the SSI estimates the in vivo biomechanical behavior of a given cornea with normal topography, adapted to each moment of the non-linear stress–strain curve [30]; (3) SP-A1 and SP-HC are considered to be the parameters that best describe the global stiffness of the eye for a given biomechanically corrected IOP [18]; and (4) whole eye movement (WEM) describes the accessory anteroposterior movement of the eyeball measured in the lateral part of the 8 mm corneal image [22]. Theoretically, a stiffer behavior is associated with lower IR values and higher SSI values [30], as well as elevated SP-A1 and SP-HC parameters [18]. Additionally, WEM is as high as the amount of energy that the cornea cannot absorb, dissipating towards the posterior pole.

In the present study, the eyes of individuals with a family history of myopia in at least one parent showed a corneal excursion that became significantly narrower over the follow-up period. While this decrease was theoretically expected with age [31], the authors hypothesize that the difference in these eyes may be related to a potential greater dissipation of energy to the posterior segment, which could be a factor in ocular elongation. However, no significant differences were found in the variation in WEM, AL, or SE during the follow-up. The authors advocate for the value of studying this hypothesis in future research. It is important to state that the average refractive error at baseline was within the range of low myopia, which is believed to be less dependent on genetic load.

The association between allergic disease and keratoconus is well-established [32]. Recently, a possible causal relationship between allergic conditions and myopia has also been investigated [33]. In the present study, individuals with a history of allergies showed significantly greater axial growth associated with a smaller increase in the SSI. Since it theoretically increases throughout life, the smaller gain in stiffness in these individuals highlights the potential pivotal role of ocular biomechanical study in the already documented [34,35] relationship between allergic disease and myopia.

There is evidence supporting the effect of orthokeratology on myopia progression; however, it remains controversial whether a mechanical mechanism alone accounts for this effect. Although no data currently exist in the literature addressing this specific hypothesis, the authors sought to explore whether a potential external mechanical pressure on the sclera during sleep—the so-called “pillow effect”—could be associated with the differences in changes in ocular biomechanical behavior, axial elongation, or myopization during the follow-up. The eyes of individuals with a predominantly ventral sleeping position also showed a smaller gain in corneo-scleral stiffness during the follow-up, with even an increase in the A1 velocity. Although less consistent as a general descriptor of corneo-scleral stiffness, this first-generation parameter is one of the most important in describing how the cornea dissipates the air puff energy, highlighting a potential pillow effect on corneal tissue remodeling in line with what has been reported in the setting of ectatic corneal disease [36]. There were also significant differences in the variation in keratometry in these eyes; however no relationship was found with axial elongation or refractive status changes. In the eyes corresponding to the preferred sleeping side, differences were observed in the variation in K2; however, no significant differences were found in any biomechanical, biometric, or refractive parameters.

Regarding eye-based analysis, a greater degree of myopization was found in the eyes with ocular surface symptoms and those with reported eye-rubbing habits, in line with recent literature [37]. Additionally, the increase in deflection amp in A1 and in the TBI in eyes with surface symptoms, as well as with the smaller increase in radius and the increase in A2 arc length in rubbed eyes, all support a lower gain in stiffness in these eyes during the follow-up. Since eye-rubbing is often associated with dry eye symptoms, the literature is scarce regarding its hypothetical relationship with myopia. The authors believe that this study, in addition to describing this association, provides evidence that it may occur through changes in ocular biomechanics.

Regarding the study of correlations, all can be found in Table 4 and Table 5, but the discussion will be focused on those considered most important to reduce the potential noise of a broader analysis.

The present study demonstrates that the more time adolescents spent engaging in outdoor activities during the follow-up, the greater the decrease in HC time and the integrated radius function and the greater the increase in the SSI, indicating altogether a greater gain in corneo-scleral stiffness. Among the lifestyle factors traditionally examined, this remains the one with the strongest evidence supporting a protective role against myopia progression [7,10]. The present study adds to the existing body of literature and is the first to provide evidence that this protective effect may be mediated by the influence of solar radiation, potentially acting as a natural corneal cross-linking (CXL) mechanism, modulating the biomechanical properties of ocular tissues [38].

The evidence is scarce regarding the association between myopia and physical activity [8,9]. In the present study, physical exercise was associated with a decrease in maximum WEM during the follow-up. This is the first description of this association in the literature. The authors conceptualize that this result may highlight the role of sports practice in stabilizing the posterior pole of the eye in response to microtraumas of the ocular surface and, consequently, in reducing axial elongation. However, in the present study, physical activity did not correlate with variations in biometric or refractive parameters.

There is growing evidence linking myopia and the use of digital devices [39,40]. In the present study, the increase in hours spent on near-vision activities (reading/writing) correlated with a greater decrease in SP-A1 overtime. Again, this is the first report of this association, and the authors believe that this may support the emmetropization theory suggesting that near-vision activates feedback mechanisms through the dopaminergic pathway [41], reducing scleral stiffness and leading to an increase in AL. However, no correlations were found between this variable and biometric or refractive variations during the follow-up. On the other hand, the amount of time spent on digital devices did not correlate with any of the main biomechanical variables.

Regarding the relationship between lifestyle parameters and the variation in biometric and refractive variables, the present study also shows that the Δ_R_VCL/AL correlates negatively with the amount of outdoor activity and positively with the time spent on digital devices. As a disproportion in the vitreous cavity growth could be related with higher degrees of myopia [42,43], the authors believe that these findings can be understood as evidence of the pivotal role of tissue biomechanics as the substrate for the relationship between lifestyle and myopization. Additionally, it highlights the role of sum-of-segments biometric approach in the study of myopia progression.

Finally, analyzing the direct relationship between variations in biomechanical parameters and biometric and refractive changes, we found the most consistent results: first, reduced myopization correlated inversely with the integrated radius function and directly with the increase in the SSI; second, greater increases in AL, VCL, and R_VCL/AL correlated negatively with the increase in the SSI. Thus, a lower overall gain in ocular stiffness during the follow-up was consistently associated with increased myopic progression in the present study, both in refractive and biometric terms.

The main strength of this study is the fact that it prospectively analyzes the modification of ocular biomechanics across 2.5 years during adolescence and its hypothetical crucial relationship with lifestyle habits and myopia progression, including a sum-of-segments biometric approach. Moreover, it encompassed the period of the COVID-19 pandemic, a period that markedly influenced lifestyle patterns known to contribute to myopia development.

The results obtained in this population with low refractive error and above childhood provide evidence for the concept of school myopia and gain greater relevance due to their potential for amplification in younger populations or those with higher degrees of myopia.

The main limitations are: (1) the inclusion of subjects of different ages, although no significant differences were found in comparative tests; (2) the execution of non-cycloplegic refraction due to restrictions imposed by the hospital’s ethics committee. However, this may add value to the results, as the greater accommodative ability at baseline could have masked some degree of myopization during follow-up; and (3) the data regarding lifestyle have some degree of subjectivity that is difficult to overcome in scientific reports.

## 5. Conclusions

This study provides novel evidence that ocular biomechanical changes during adolescence are closely linked to myopia progression and influenced by lifestyle factors. A lower gain in corneo-scleral stiffness was consistently associated with greater axial elongation and refractive myopization, while outdoor activity appeared protective, and near-work and ocular surface symptoms and eye-rubbing showed adverse effects. These findings suggest that ocular biomechanics may represent the physiological bridge between the environmental exposure and myopization, highlighting their potential role in future strategies for early detection and prevention of myopia.

## Figures and Tables

**Table 1:** Scheimpflug camera-derived corneal biomechanical parameters with explanation and abbreviations.

Parameters	Abbreviations	Explanation	Anatomical Unit Movement	Theoretical Meaning of Higher Values
cIOP [mmHg]	cIOP	Corvis-derived intraocular pressure	Non applied	Non applied
cPachy [µm]	cCCT	Corvis-derivated central corneal thickness	Non applied	Non applied
**1st Generation Parameters**		**Explanation**	**Anatomical Unit Movement**	**Theoretical Meaning of Higher Values**
Deformation Amp. Max [mm]	MaxDefoA	Corneal deformation amplitude during MaxDT, as the sum of corneal deflection amplitude and MaxWEM	Ocular deformation	Less rigid behavior
A1 Time [ms]	A1T	Time from the measurement beginning to the first applanation moment	Corneal deflection	More rigid behavior
A1 Velocity [m/s]	A1V	Velocity of the corneal apex during the first applanation	Corneal deflection	Less rigid behavior
A2 Time [ms]	A2T	Time from the measurement beginning to the second applanation moment	Corneal deflection	Less rigid behavior
A2 Velocity [m/s]	A2V	Velocity of the corneal apex during the second applanation	Corneal deflection	Less rigid behavior
HC Time [ms]	HCT	Time from the measurement beginning to the moment of reaching the highest concavity (HC)	Corneal deflection	Less rigid behavior
Peak Dist. [mm]	HCPD	Distance between the corneal peaks at the HC	Corneal deflection	Less rigid behavior
Radius [mm]	HCR	Radius of corneal curvature during the HC	Corneal deflection	More rigid behavior
A1 Deformation Amp. [mm]	A1DefoA	Corneal deformation amplitude during A1, as the sum of corneal deflection amplitude and MaxWEM	Ocular deformation	Less rigid behavior
HC Deformation Amp. [mm]	HCDefoA	Corneal deformation amplitude during HC, as the sum of corneal deflection amplitude and MaxWEM	Ocular deformation	Less rigid behavior
A2 Deformation Amp. [mm]	A2DefoA	Corneal deformation amplitude during A2, as the sum of corneal deflection amplitude and MaxWEM	Ocular deformation	Less rigid behavior
A1 Deflection Length [mm]	A1DL	Horizontal length of the flattened cornea at the A1	Corneal deflection	More rigid behavior
HC Deflection Length [mm]	HCDL	Horizontal length of the flattened cornea at the HC	Corneal deflection	More rigid behavior
A2 Deflection Length [mm]	A2DL	Horizontal length of the flattened cornea at the A2	Corneal deflection	More rigid behavior
A1 Deflection Amp. [mm]	A1DA	Corneal deflection amplitude during A1, determined as the displacement of the corneal apex in relation to the initial state without the MaxWEM quantification	Corneal deflection	Less rigid behavior
HC Deflection Amp. [mm]	HCDA	Corneal deflection amplitude during HC, determined as the displacement of the corneal apex in relation to the initial state without the MaxWEM quantification	Corneal deflection	Less rigid behavior
A2 Deflection Amp. [mm]	A2DA	Corneal deflection amplitude during A2, determined as the displacement of the corneal apex in relation to the initial state without the MaxWEM quantification	Corneal deflection	Less rigid behavior
Deflection Amp. Max [mm]	MaxDA	Corneal deflection amplitude during MaxDT	Corneal deflection	Less rigid behavior
Deflection Amp. Max [ms]	MaxDT	Moment of the maximum corneal deflection, during the oscillatory phase near HC	Corneal deflection	Less rigid behavior
Whole Eye Movement Max [mm]	MaxWEM	Amplitude of the Maximum whole eye movement	Ocular deformation	More energy to posterior pole
Whole Eye Movement Max [ms]	MaxWEMT	Time at which occurs the amplitude of the Maximum whole eye movement (near A2)	Ocular deformation	Less energy to posterior pole
A1 Deflection Area [mm^2^]	A1DArea	Deflection area in A1	Corneal deflection	Less rigid behavior
HC Deflection Area [mm^2^]	HCDArea	Deflection area in HC	Corneal deflection	Less rigid behavior
A2 Deflection Area [mm^2^]	A2DArea	Deflection area in A2	Corneal deflection	Less rigid behavior
A1 dArc Length [mm]	A1dArcL	Delta arc length of corneal surface in A1	Corneal deflection	Less rigid behavior
HC dArc Length [mm]	HCdArcL	Delta arc length of corneal surface in HC	Corneal deflection	Less rigid behavior
A2 dArc Length [mm]	A2dArcL	Delta arc length of corneal surface in A2	Corneal deflection	Less rigid behavior
dArcLengthMax [mm]	MaxdArcL	Delta arc length of corneal surface in MaxDT	Corneal deflection	Less rigid behavior
**2nd Generation Parameters**		**Explanation**	**Anatomical Unit Movement**	**Theoretical Meaning of Higher Values**
Max InverseRadius [mm^−1^]	MIR	1/HCR	Corneal deflection	Less rigid behavior
DA Ratio Max (2 mm)	DARM2	Ápex MaxDA/MaxDA at 2 mm from the ápex	Corneal deflection	Less rigid behavior
PachySlope [µm]	PqS	Peripheric (8 mm horizontal) pachymetry/Ápex pachymetry	Non applied	Non applied
DA Ratio Max (1 mm)	DARM1	Ápex MaxDA/MaxDA at 1 mm from the ápex	Corneal deflection	Less rigid behavior
Ambrosio Relational Thickness (8 mm)	ARTh	Ambrosio Relational Thickness within the horizontal 8 mm cornea of the image	Non applied	Non applied
Biomechanically-corrected IOP	bIOP	IOP adjusted for biomechanical parameters	Non applied	Non applied
Integrated Radius [mm^−1^]	IR	Area under the curve of the 1/HCR function	Corneal deflection	Less rigid behavior
Stiffness parameter in A1	SP-A1	Air puff pressure − bIOP/A1DA	Ocular deformation	More rigid behavior
Corvis biomechanical index	CBI	Exponential function score made through a logistic regression analysis of 6 parameters (SP-A1, DARM1, DARM2, ARTh, A1V and MaxDefoA) and adjusted for IOP and CCT to describe ectasia risk	Corneal deflection	Less rigid behavior
Tomographic and Biomechanical Index	TBI	Algorithm including tomographic and biomechanical parameters for the discrimination of eyes with corneal ectasia susceptibility	Corneal deflection	Less rigid behavior
Stress Strain Index	SS-I	Finite element modeling algorithm for the estimation of the non-linear in vivo biomechanical behaviour in corneal with normal topography	Ocular deformation	More rigid behavior
A1 DeflectionVelocity [m/s]	A1DV	Velocity of the corneal deflection during the first applanation	Corneal deflection	Less rigid behavior
A2 DeflectionVelocity [m/s]	A2DV	Velocity of the corneal deflection during the second applanation	Corneal deflection	Less rigid behavior
Corrected Corvis biomechanical index	cCBI	Adjusted CBI for post queretorrefractive surgery corneas	Corneal deflection	Less rigid behavior
Biomechanically-corrected IOP (2nd version)	bIOP2	2nd version of bIOP	Non applied	Non applied
Stress Strain Index (2nd version)	SS-II	2nd version of SSI-I	Ocular deformation	More rigid behavior
Stiffness parameter in HC	SP-HC	Air puff pressure − bIOP/HCDA	Ocular deformation	More rigid behavior
Stiffness parameter in MaxDT	SP-MAxDA	Air puff pressure − bIOP/MaxDA	Ocular deformation	More rigid behavior

**Table 2:** Per-individual analysis (63 individuals).

	Family History of Myopia					Alergies History					Predominantly Ventral Sleeping				
	Yes (n = 24)		No (n = 39)		*p*	Yes (n = 28)		No (n = 35)		*p*	Yes (n = 38)		No (n = 25)		*p*
	Mean	SD	Mean	SD		Mean	SD	Mean	SD		Mean	SD	Mean	SD	
Age at baseline (years)	14.436	2.673	13.856	2.574	0.78	13.939	2.639	14.27	2.655	0.488	13.806	2.55	14.535	2.691	0.13

Δ_AL (mm)	0.1	0.102	0.136	0.2	0.8	0.155	0.206	0.087	0.094	0.032 *	0.124	0.186	0.12	0.143	0.71
Δ_VCL (mm)	0.076	0.097	0.101	0.199	0.849	0.112	0.206	0.068	0.096	0.35	0.097	0.186	0.084	0.135	0.463
Δ_Ratio VCL/AL	2.607 × 10^−4^	0.002	1.071 × 10^−4^	0.003	0.479	7.398 × 10^−5^	0.003	3.265 × 10^−4^	0.002	0.213	2.840 × 10^−4^	0.003	−15.88	0.002	0.363

Δ_SE (D)	−0.235	0.832	−0.375	0.893	0.383	−0.361	0.973	−0.271	0.742	0.236	−0.253	1.012	−0.428	0.569	0.138

Δ_K1 (D)	0.025	0.183	0.01	0.208	0.224	−0.024	0.159	0.028	0.224	0.152	0.038	0.196	−0.05	0.194	0.015 *
Δ_K2 (D)	0.025	0.383	0.012	0.275	0.241	0.07	0.232	−0.025	0.378	0.106	0.031	0.356	0.09	0.248	0.039 *

Δ_cPachy [µm]	−9.458	13.577	−8.973	14.48	0.853	−10.833	15.263	−7.924	13.135	0.264	−7.708	14.306	−11.26	13.608	0.172
Δ_Deformation AmpΔ_ Max [mm]	−0.008	0.08	0.001	−0.014	0.002 **	−0.014	0.07	0.011	0.09	0.102	0.003	0.083	−0.01	0.083	0.406
Δ_A1 Time [ms]	0.122	0.258	0.086	0.222	0.42	0.102	0.184	0.091	0.274	0.627	0.101	0.26	0.097	0.201	0.853
Δ_A1 Velocity [m/s]	0.013	0.016	0.003	0.014	0.099	−0.01	0.012	0.004	0.017	0.159	0.004	0.016	−0.002	0.013	0.046 *
Δ_A2 Time [ms]	−0.014	0.074	0.037	0.056	0.045 *	0.031	0.311	0.016	0.574	0.41	0.026	0.548	0.005	0.332	0.141
Δ_A2 Velocity [m/s]	0.013	0.029	0.013	0.054	0.483	0.009	0.022	0.015	0.058	0.991	0.012	0.054	0.014	0.031	0.225
Δ_HC Time [ms]	0.001	0.007	0.001	0.009	0.692	0.097	0.608	0.005	0.681	0.256	0.076	0.706	−0.021	0.555	0.527
Δ_Peak DistΔ_ [mm]	−0.01	0.23	0.009	0.236	0.414	−0.034	0.163	0.039	0.272	0.059	0.003	0.259	−0.002	0.191	0.468
Δ_Radius [mm]	0.329	0.663	0.234	0.616	0.463	0.208	0.597	0.325	0.667	0.333	0.265	0.686	0.279	0.557	0.745
Δ_A1 Deformation AmpΔ_ [mm]	−0.002	0.016	7.432 × 10^−4^	0.014	0.409	−0.003	0.015	0.002	0.014	0.087	1.944 × 10^−4^	0.018	0.005	0.009	0.969
Δ_HC Deformation AmpΔ_ [mm]	−0.008	0.08	0.001	0.085	0.559	−0.014	0.07	0.011	0.09	0.102	0.003	0.083	−0.01	0.083	0.406
Δ_A2 Deformation AmpΔ_ [mm]	0.007	0.062	0.018	0.059	0.536	0.015	0.068	0.013	0.054	0.627	0.024	0.069	2.933 × 10^−19^	0.041	0.069
Δ_A1 Deflection Length [mm]	0.003	0.126	0.008	0.287	0.996	0.008	0.147	0.004	0.296	0.797	0.015	0.287	−0.006	0.146	0.458
Δ_HC Deflection Length [mm]	−0.091	0.436	−0.013	0.406	0.328	−0.103	0.331	0.018	0.478	0.125	−0.02	0.463	−0.075	0.352	0.484
Δ_A2 Deflection Length [mm]	−0.131	0.546	−0.177	0.67	0.487	−0.1	0.7	−0.194	0.554	0.873	−0.17	0.571	−0.145	0.694	0.912
Δ_A1 Deflection AmpΔ_ [mm]	−0.002	0.017	3.784 × 10^−4^	0.009	0.234	−0.002	0.008	0.002	0.016	0.233	7.361 × 10^−4^	0.015	−0.001	0.008	0.9
Δ_HC Deflection AmpΔ_ [mm]	−0.002	2.459	−0.003	0.08	0.147	−0.023	0.054	−0.025	2.101	0.159	−0.045	2.01	−0.012	0.077	0.717
Δ_A2 Deflection AmpΔ_ [mm]	−0.005	0.017	0.003	0.055	0.23	−0.005	0.012	0.005	0.059	0.58	0.004	0.057	−0.005	0.013	0.405
Δ_Deflection AmpΔ_ Max [mm]	−0.002	0.018	0.005	0.095	0.186	−0.015	0.055	−0.064	5.294	0.49	−0.094	5.068	−0.014	0.081	0.233
Δ_Deflection AmpΔ_ Max [ms]	−0.087	1.243	−0.092	0.868	0.38	−0.044	0.922	−0.09	1.119	0.145	−0.164	1.053	−0.279	1.008	0.855
Δ_Whole Eye Movement Max [mm]	0.01	0.064	0.011	0.074	0.844	0.02	0.068	0.003	0.072	0.249	0.014	0.084	0.005	0.041	0.281
Δ_Whole Eye Movement Max [ms]	0.086	0.84	0.323	1.599	0.548	0.187	0.775	0.285	1.702	0.87	0.256	1.664	0.192	0.709	0.628
Δ_A1 Deflection Area [mm^2^]	0.007	0.039	0.015	0.025	0.296	−0.002	0.026	0.006	0.036	0.344	0.006	0.034	−0.003	0.027	0.146
Δ_HC Deflection Area [mm^2^]	−0.029	0.507	−0.018	0.55	0.241	−0.089	0.282	0.045	0.668	0.113	−0.024	0.611	−0.02	0.396	0.641
Δ_A2 Deflection Area [mm^2^]	−0.016	0.05	0.002	0.173	0.857	−0.022	0.056	0.011	0.181	0.316	0.003	0.175	−0.016	0.053	0.537
Δ_A1 dArc Length [mm]	7.083 × 10^−4^	0.004	0.016	0.004	0.588	5.926 × 10^−4^	0.004	1.434 × 10^−4^	0.004	0.394	1.389 × 10^−4^	0.004	1.600 × 10^−4^	0.003	0.811
Δ_HC dArc Length [mm]	−0.001	0.025	0.016	0.147	0.43	0.002	0.018	0.015	0.156	0.292	0.013	0.15	0.003	0.015	0.049 *
Δ_A2 dArc Length [mm]	0.003	0.009	0.005	0.035	0.29	0.002	0.008	0.006	0.038	0.929	0.006	0.036	0.001	0.006	0.771
Δ_dArcLengthMax [mm]	−0.005	0.025	−0.005	0.021	0.935	−0.003	0.02	−0.007	0.024	0.523	−0.008	0.023	−0.007	0.022	0.533
Δ_Max InverseRadius [mm^−1^]	−0.013	0.03	5.541 × 10^−4^	0.053	0.17	−0.007	0.011	−0.003	0.061	0.922	−0.005	0.059	−0.005	0.012	0.182
Δ_DA Ratio Max (2 mm)	−0.093	0.344	−0.037	0.243	0.132	−0.041	0.246	−0.07	0.321	0.802	−0.092	0.334	−0.011	0.195	0.117
Δ_PachySlope [µm]	1.303	4.253	1.361	4.653	0.945	1.469	4.959	1.371	4.082	0.906	0.705	4.341	2.249	4.568	0.061
Δ_DA Ratio Max (1 mm)	−0.002	0.038	−0.002	0.06	0.455	−0.004	0.042	−0.012	0.06	0.558	−0.009	0.063	−0.007	0.032	0.767
Δ_Ambrosio Relational Thickness	−2.898	1.354	−3.127	1.684	0.217	−8.979	1.789	−7.885	1.634	0.446	−9.854	1.103	−8.5	1.335	0.389
Δ_Biomechanically-corrected IOP	1.019	1.9	0.714	1.684	0.512	0.869	1.487	0.758	1.988	0.525	0.831	1.947	0.838	1.501	0.927
Δ_Integrated Radius [mm^−1^]	−0.644	0.036	−0.232	0.068	0.006 **	−0.402	0.604	−0.367	1.029	0.5	−0.393	0.974	−0.395	0.668	0.777
Δ_Stiffness parameter in A1	5.088	14.071	0.793	12.465	0.08	3.708	10.579	3.906	14.865	0.275	3.134	13.91	1.506	12.254	0.529
Δ_Corvis biomechanical index	−0.031	0.201	−0.01	0.159	0.374	−0.022	0.204	−0.014	0.153	0.75	−0.043	0.169	0.018	0.18	0.027 *
Δ_Tomographic and Biomechanical Index	−0.005	0.126	0.012	0.181	0.473	0.045	0.162	−0.019	0.15	0.198	0.004	0.16	0.007	0.166	0.758
Δ_Stress Strain Index	0.063	0.103	0.026	0.132	0.105	0.012	0.1	0.07	0.133	0.01 **	0.032	0.131	0.052	0.109	0.37
Δ_Biomechanically-corrected IOP (2nd)	0.4275	23.776	0.441	1.807	0.263	0.844	1.483	0.843	1.316	0.3	0.821	19.494	0.694	1.561	0.679
Δ_Stress Strain Index (2nd version)	0.028	0.219	0.049	0.066	0.552	0.046	0.063	0.037	0.091	0.337	0.033	0.184	0.052	0.058	0.817
Δ_Stiffness parameter in HC	0.84	3.03	0.151	2.249	0.152	0.487	2.027	0.279	2.98	0.498	0.347	2.86	0.531	2.181	0.56

Footnotes: AL: Axial Length; VCL: Vitreous Cavity Length; Δ: delta of progression; SE: Spherical Equivalent; K1: keratometry in the flat meridian; K2: Keratometry in the steep meridian; Δ: Delta of variation between baseline and the end of follow-up; * *p* ≤ 0.05; ** *p* ≤ 0.01.

**Table 3:** Per-Eye analysis (126 eyes).

	Ipsilateral Sleeping Position					Dry eye Symptoms					Eye-Rubbing Habits				
	Yes (n = 52)		No (n = 74)		*p*	Yes (n = 57)		No (n = 69)		*p*	Yes (n = 84)		No (n = 42)		*p*
	Mean	SD	Mean	SD		Mean	SD	Mean	SD		Mean	SD	Mean	SD	
Age at baseline (years)	14.236	2.573	13.546	2.674	0.213	14.532	2.502	13.890	2.579	0.176	14.418	2.722	13.549	2.303	0.111

Δ_AL (mm)	0.117	0.168	0.126	0.172	0.753	0.13	0.184	0.118	0.16	0.693	0.124	0.169	0.119	0.173	0.533
Δ_VCL (mm)	0.085	0.167	0.096	0.168	0.966	0.099	0.18	0.084	0.159	0.631	0.095	0.168	0.084	0.167	0.657
Δ_Ratio VCL/AL	8.337 × 10^−5^	0.002	2.250 × 10^−4^	0.002	0.64	2.908 × 10^−4^	0.002	−32.66	0.002	0.694	3.104 × 10^−4^	0.002	−16.96	0.002	0.807

Δ_SE (D)	−0.38	0.965	−0.28	0.8	0.273	−0.485	0.812	−0.173	0.901	0.049 *	−0.414	0.787	−0.125	1.003	0.04 *

Δ_K1 (D)	0.024	0.203	−0.014	0.196	0.282	0.014	0.184	−0.005	0.211	0.604	−0.017	0.184	0.008	0.23	0.845
Δ_K2 (D)	0.071	0.373	0.03	0.282	0.031 *	0	0.394	0.038	0.25	0.521	0.014	0.365	0.025	0.205	0.863

Δ_cPachy [µm]	−10.08	14.723	−8.528	13.678	0.551	−9.214	13.817	−9.313	14.399	0.97	−10.22	14.722	−7	12.549	0.237
Δ_Deformation AmpΔ_ Max [mm]	0.003	0.079	−0.006	0.086	0.563	−0.013	0.087	0.009	0.08	0.152	0.005	0.085	−0.017	0.077	0.173
Δ_A1 Time [ms]	0.091	0.243	0.106	0.234	0.892	0.148	0.242	0.06	0.229	0.107	0.104	0.252	0.091	0.204	0.95
Δ_A1 Velocity [m/s]	0.001	0.013	0.001	0.016	0.777	9.107 × 10^−4^	0.013	0.002	0.017	0.877	0.001	0.014	0.001	0.016	0.825
Δ_A2 Time [ms]	0.055	0.277	−0.059	0.564	0.697	0.059	0.312	−0.021	0.576	0.69	0.052	0.52	0.047	0.346	0.751
Δ_A2 Velocity [m/s]	0.011	0.028	0.014	0.055	0.926	0.012	0.028	0.014	0.057	0.606	0.015	0.051	0.008	0.031	0.664
Δ_HC Time [ms]	0.122	0.733	−0.023	0.58	0.395	0.111	0.747	−0.039	0.549	0.432	0.082	0.654	−0.057	0.633	0.207
Δ_Peak DistΔ_ [mm]	0.012	0.217	−0.007	0.244	0.925	−0.022	0.227	0.024	0.239	0.191	−0.008	0.253	0.02	0.186	0.729
Δ_Radius [mm]	0.192	0.434	0.326	0.74	0.108	0.361	0.607	0.213	0.647	0.258	0.193	0.691	0.432	0.463	0.018 *
Δ_A1 Deformation AmpΔ_ [mm]	7.400 × 10^−4^	0.011	0.004	0.017	0.517	0.001	0.013	0.007	0.014	0.343	−0.019	0.016	2.250 × 10^−4^	0.013	0.933
Δ_HC Deformation AmpΔ_ [mm]	0.003	0.079	−0.006	0.086	0.563	−0.013	0.087	0.009	0.08	0.152	0.005	0.085	−0.017	0.077	0.173
Δ_A2 Deformation AmpΔ_ [mm]	0.016	0.056	0.012	0.063	0.584	0.021	0.054	0.01	0.063	0.206	0.016	0.062	0.009	0.057	0.384
Δ_A1 Deflection Length [mm]	−0.036	0.277	0.037	0.202	0.154	0.037	0.306	−0.019	0.153	0.032 *	0.006	0.266	0.007	0.172	0.881
Δ_HC Deflection Length [mm]	−0.034	0.425	−0.051	0.416	0.827	−0.062	0.428	−0.024	0.414	0.628	−0.063	0.437	−0.006	0.382	0.488
Δ_A2 Deflection Length [mm]	−0.093	0.669	−0.207	0.589	0.671	−0.138	0.69	−0.177	0.567	0.847	−0.198	0.566	−0.085	0.723	0.756
Δ_A1 Deflection AmpΔ_ [mm]	0.002	0.008	3.611 × 10^−4^	0.015	0.817	0.002	0.008	−0.018	0.016	0.031 *	4.268 × 10^−4^	0.014	−0.011	0.009	0.87
Δ_HC Deflection AmpΔ_ [mm]	−0.008	0.079	−0.247	2.01	0.952	−0.021	0.078	−0.266	2.133	0.241	−0.216	1.884	−0.012	0.073	0.821
Δ_A2 Deflection AmpΔ_ [mm]	−0.003	0.01	0.002	0.057	0.877	−0.002	0.012	0.002	0.06	0.133	0.002	0.053	−0.004	0.013	0.94
Δ_Deflection AmpΔ_ Max [mm]	−0.005	0.078	−0.006	0.088	0.698	−0.016	0.075	−0.066	0.054	0.458	−0.026	0.049	−0.01	0.068	0.627
Δ_Deflection AmpΔ_ Max [ms]	−0.219	1.094	−0.206	0.994	0.821	−0.269	1.046	−0.148	1.03	0.319	−0.222	1.095	−0.19	0.901	0.787
Δ_Whole Eye Movement Max [mm]	0.009	0.083	0.012	0.06	0.517	0.016	0.082	0.007	0.057	0.203	0.016	0.059	1.000 × 10^−4^	0.087	0.298
Δ_Whole Eye Movement Max [ms]	0.367	1.669	0.134	1.083	0.375	0.172	0.76	0.289	1.706	0.791	0.337	0.866	0.624	1.97	0.33
Δ_A1 Deflection Area [mm^2^]	8.400 × 10^−4^	0.024	0.004	0.036	0.747	0.006	0.025	0.002	0.034	0.206	0.005	0.035	−0.002	0.023	0.623
Δ_HC Deflection Area [mm^2^]	0.053	0.431	−0.04	0.594	0.921	−0.045	0.427	0.333	0.617	0.374	−0.029	0.601	−0.01	0.355	0.711
Δ_A2 Deflection Area [mm^2^]	−0.015	0.046	0.002	0.176	0.731	−0.012	0.052	0.005	0.183	0.76	0.016	0.165	−0.015	0.053	0.9
Δ_A1 dArc Length [mm]	6.000 × 10^−5^	0.003	2.083 × 10^−4^	0.004	0.793	0.005	0.003	2.031 × 10^−4^	0.004	0.348	3.049 × 10^−4^	0.004	0.003	0.004	0.672
Δ_HC dArc Length [mm]	−0.001	0.018	0.016	0.149	0.801	−0.004	0.023	0.02	0.157	0.106	0.015	0.14	−0.002	0.016	0.478
Δ_A2 dArc Length [mm]	0.002	0.005	0.005	0.036	0.2	9.091 × 10^−4^	0.005	0.006	0.038	0.76	0.007	0.034	−0.001	0.008	0.032 *
Δ_dArcLengthMax [mm]	−0.004	0.02	−0.006	0.024	0.99	−0.008	0.023	−0.003	0.022	0.176	−0.007	0.024	−0.008	0.019	0.156
Δ_Max InverseRadius [mm^−1^]	−0.01	0.028	−0.005	0.054	0.745	−0.009	0.011	−0.001	0.062	0.121	−0.004	0.055	−0.006	0.013	0.77
Δ_DA Ratio Max (2 mm)	−0.051	0.231	−0.065	0.322	0.63	−0.064	0.254	−0.065	0.307	0.791	−0.083	0.296	−0.01	0.264	0.181
Δ_PachySlope [µm]	1.055	4.448	1.535	4.526	0.563	1.107	4.255	1.189	4.181	0.488	1.409	4.453	1.193	4.594	0.804
Δ_DA Ratio Max (1 mm)	−0.008	0.042	−0.008	0.059	0.668	−0.014	0.049	−0.006	0.053	0.521	−0.004	0.053	−0.016	0.051	0.643
Δ_Ambrosio Relational Thickness	−27.219	147.623	−23.244	136.698	0.69	−35.009	152.71	−15.998	127.381	0.486	−26.66	141.207	−21.209	141.344	0.693
Δ_Biomechanically-corrected IOP	0.768	1.827	0.879	1.743	0.743	0.443	1.897	0.564	1.649	0.167	0.917	1.863	0.662	1.573	0.659
Δ_Integrated Radius [mm^−1^]	−0.5	0.897	−0.32	0.83	0.49	−0.435	0.705	−0.37	0.987	0.292	−0.405	0.93	−0.37	0.7	0.933
Δ_Stiffness parameter in A1	1.007	13.356	3.486	13.125	0.237	1.553	12.905	2.643	13.316	0.323	2.362	13.662	2.661	12.45	0.677
Δ_Corvis biomechanical index	−0.023	0.21	−0.015	0.147	0.774	−0.017	0.175	−0.019	0.179	0.851	−0.016	0.168	−0.022	0.192	0.655
Δ_Tomographic and Biomechanical Index	0.02	0.175	−0.005	0.153	0.526	0.057	0.168	−0.04	0.146	<0.001 ***	0.008	0.145	−0.061	0.194	0.742
Δ_Stress Strain Index	0.049	0.113	0.034	0.129	0.504	0.053	0.121	0.031	0.125	0.342	0.038	0.111	0.044	0.145	0.817
Δ_Biomechanically-corrected IOP (2nd)	0.674	1.911	0.299	1.471	0.805	0.911	1.978	0.888	1.663	0.27	0.724	1.249	0.362	1.674	0.467
Δ_Stress Strain Index (2nd version)	0.045	0.051	0.038	0.186	0.221	0.059	0.068	0.027	0.19	0.36	0.029	0.174	0.065	0.046	0.096
Δ_Stiffness parameter in HC	0.219	2.378	0.563	2.743	0.901	0.752	2.402	0.137	2.764	0.205	0.421	2.777	0.424	2.207	0.777

Footnotes: AL: Axial Length; VCL: Vitreous Cavity Length; Δ: delta of progression; SE: Spherical Equivalent; K1: keratometry in the flat meridian; K2: Keratometry in the steep meridian; Δ: Delta of variation between baseline and the end of follow-up; * *p* ≤ 0.05; *** *p* ≤ 0.001.

**Table 4:** Correlations between biomechanical parameters and lifestyle variables.

	Weekly Hours of Outdoor Activity				Weekly Hours of Physical Activity				Daily Hours Spent on Digital Devices				Daily Hours Spent Reading/Writing			
	Pearson		Spearman		Pearson		Spearman		Pearson		Spearman		Pearson		Spearman	
	*r*	*p*	*rho*	*p*	*r*	*p*	*rho*	*p*	*r*	*p*	*rho*	*p*	*r*	*p*	*rho*	*p*
Age at baseline	0.137	0.127	0.163	0.068	−0.046	0.613	−0.094	0.296	0.194	0.029 *	0.203	0.022 *	−0.12	0.182	−0.118	0.188
Δ_cPachy [µm]	−0.171	0.059	−0.17	0.061	−0.011	0.901	0.031	0.734	−0.105	0.25	−0.083	0.365	−0.239	0.008 **	−0.207	0.022 *
Δ_Deformation AmpΔ_ Max [mm]	−0.152	0.096	−0.186	0.041 *	−0.194	0.032 *	−0.176	0.052	0.097	0.288	0.086	0.347	0.11	0.226	0.118	0.197
Δ_A1 Time [ms]	0.138	0.129	0.109	0.231	0.053	0.562	0.139	0.126	−0.065	0.479	−0.079	0.388	−0.002	0.985	−0.012	0.892
Δ_A1 Velocity [m/s]	−0.085	0.353	−0.101	0.27	0.086	0.344	0.093	0.309	0.105	0.248	0.097	0.29	0.12	0.187	0.113	0.214
Δ_A2 Time [ms]	−0.169	0.065	−0.209	0.022 *	−0.071	0.444	−0.105	0.254	0.123	0.182	0.149	0.104	0.268	0.003 **	0.26	0.004 **
Δ_A2 Velocity [m/s]	0.09	0.329	0.061	0.506	0.072	0.433	0.086	0.352	−0.04	0.663	0.01	0.915	−0.085	0.356	−0.004	0.969
Δ_HC Time [ms]	−0.209	0.021 *	−0.21	0.02 *	0.059	0.521	0.063	0.49	0.03	0.739	0.042	0.649	−0.009	0.925	−0.04	0.66
Δ_Peak DistΔ_ [mm]	−0.097	0.288	−0.09	0.322	−0.056	0.538	−0.111	0.224	0.08	0.383	0.028	0.762	0.133	0.144	0.106	0.246
Δ_Radius [mm]	0.085	0.351	0.107	0.242	0.022	0.807	0.078	0.394	−0.045	0.626	−0.009	0.921	0.006	0.948	−0.026	0.773
Δ_A1 Deformation AmpΔ_ [mm]	0.004	0.964	−0.052	0.571	3.832 × 10^−4^	0.997	0.042	0.65	0.076	0.408	0.049	0.596	0.045	0.619	0.064	0.487
Δ_HC Deformation AmpΔ_ [mm]	−0.152	0.096	−0.186	0.041 *	−0.194	0.032 *	−0.176	0.052	0.097	0.288	0.086	0.347	0.11	0.226	0.118	0.197
Δ_A2 Deformation AmpΔ_ [mm]	0.009	0.921	−0.077	0.403	−0.06	0.513	0.009	0.925	−0.002	0.981	−0.007	0.94	0.117	0.202	0.132	0.149
Δ_A1 Deflection Length [mm]	0.006	0.95	1.927 × 10^−4^	0.998	0.082	0.372	0.11	0.231	−0.039	0.676	−0.003	0.974	−0.036	0.692	−0.003	0.972
Δ_HC Deflection Length [mm]	−0.135	0.148	−0.124	0.182	0.041	0.661	−0.039	0.678	0.057	0.542	0.041	0.665	0.016	0.867	0.014	0.885
Δ_A2 Deflection Length [mm]	−0.008	0.93	−0.027	0.769	−0.026	0.777	0.006	0.946	−0.095	0.309	−0.092	0.322	0.125	0.18	0.096	0.303
Δ_A1 Deflection AmpΔ_ [mm]	0.027	0.771	−0.007	0.942	0.121	0.186	0.127	0.164	0.101	0.27	0.045	0.623	0.057	0.532	0.057	0.534
Δ_HC Deflection AmpΔ_ [mm]	−0.078	0.394	−0.163	0.072	−0.096	0.291	−0.157	0.085	−0.159	0.08	0.006	0.949	−0.069	0.453	0.074	0.416
Δ_A2 Deflection AmpΔ_ [mm]	0.036	0.698	−0.09	0.328	0.054	0.556	0.065	0.477	−0.083	0.367	−0.054	0.556	−0.055	0.549	0.182	0.047 *
Δ_Deflection AmpΔ_ Max [mm]	−0.073	0.425	−0.121	0.186	−0.093	0.309	−0.172	0.058	−0.161	0.076	0.027	0.767	−0.072	0.429	0.062	0.499
Δ_Deflection AmpΔ_ Max [ms]	−0.007	0.935	−0.088	0.336	0.022	0.808	−0.036	0.693	0.083	0.361	0.055	0.545	−0.04	0.66	−0.033	0.715
Δ_Whole Eye Movement Max [mm]	−0.144	0.113	−0.142	0.12	−0.218	0.016 *	−0.096	0.295	0.111	0.223	0.06	0.508	0.11	0.228	0.114	0.21
Δ_Whole Eye Movement Max [ms]	−0.011	0.908	0.005	0.957	0.043	0.636	−0.025	0.783	0.157	0.083	0.127	0.163	−0.017	0.854	0.034	0.711
Δ_A1 Deflection Area [mm^2^]	0.05	0.582	0.003	0.978	0.192	0.034 *	0.203	0.025 *	0.158	0.083	0.077	0.402	−0.003	0.971	−0.038	0.681
Δ_HC Deflection Area [mm^2^]	−0.061	0.504	−0.109	0.234	−0.063	0.492	−0.129	0.157	0.157	0.084	0.091	0.317	0.184	0.043 *	0.148	0.104
Δ_A2 Deflection Area [mm^2^]	0.062	0.5	−0.056	0.545	0.052	0.576	0.036	0.699	−0.084	0.359	−0.016	0.865	−0.117	0.201	−0.019	0.833
Δ_A1 dArc Length [mm]	−0.057	0.53	−0.011	0.905	−0.129	0.158	−0.142	0.118	0.138	0.13	0.155	0.089	−0.091	0.32	−0.1	0.272
Δ_HC dArc Length [mm]	0.069	0.451	0.013	0.885	0.011	0.903	−0.07	0.443	−0.102	0.265	−0.099	0.276	−0.135	0.138	−0.106	0.243
Δ_A2 dArc Length [mm]	0.064	0.485	0.041	0.655	0.078	0.398	0.074	0.42	−0.062	0.5	0.062	0.503	−0.124	0.176	−0.074	0.424
Δ_dArcLengthMax [mm]	0.031	0.737	0.059	0.515	0.069	0.451	0.123	0.178	−0.202	0.026 *	−0.184	0.042 *	−0.138	0.131	−0.122	0.181
Δ_Max InverseRadius [mm^−1^]	−0.03	0.742	−0.233	0.01 **	−0.046	0.617	−0.122	0.179	−0.128	0.16	0.003	0.974	−0.108	0.237	0.105	0.249
Δ_DA Ratio Max (2 mm)	−0.083	0.365	−0.044	0.63	−0.078	0.392	−0.112	0.221	−0.044	0.63	0.029	0.748	0.1	0.273	0.132	0.148
Δ_PachySlope [µm]	−0.064	0.486	−0.055	0.549	0.031	0.732	0.008	0.928	0.147	0.107	0.135	0.137	−0.028	0.757	−0.017	0.855
Δ_DA Ratio Max (1 mm)	0.039	0.671	0.068	0.458	0.044	0.631	−0.004	0.965	0.008	0.931	0.014	0.878	−0.081	0.373	−0.069	0.449
Δ_Ambrosio Relational Thickness	0.06	0.513	0.018	0.843	−0.093	0.308	−0.055	0.544	0.017	0.854	−0.029	0.755	−0.121	0.183	−0.027	0.765
Δ_Biomechanically-corrected IOP	0.161	0.077	0.131	0.15	0.051	0.576	0.146	0.109	−0.032	0.724	−0.063	0.493	−0.011	0.906	−0.037	0.683
Δ_Integrated Radius [mm^−1^]	−0.202	0.026 *	−0.264	0.003 **	−0.072	0.433	−0.09	0.326	0.013	0.891	0.094	0.302	0.06	0.509	0.147	0.106
Δ_Stiffness parameter in A1	0.109	0.236	0.083	0.363	0.032	0.728	0.022	0.81	−0.087	0.341	−0.13	0.156	−0.245	0.007 **	−0.283	0.002 **
Δ_Corvis biomechanical index	0.011	0.906	0.065	0.482	−0.076	0.416	−0.056	0.549	0.078	0.404	0.049	0.596	0.074	0.425	0.081	0.383
Δ_Tomographic and Biomechanical Index	0.104	0.264	0.111	0.233	−0.092	0.32	−0.132	0.154	−0.007	0.942	−0.009	0.924	0.128	0.168	0.155	0.093
Δ_Stress Strain Index	0.209	0.022 *	0.239	0.008 **	0.122	0.183	0.151	0.098	−0.079	0.386	−0.054	0.557	−0.079	0.391	−0.081	0.38
Δ_Biomechanically-corrected IOP (2nd)	0.086	0.344	0.101	0.268	0.098	0.285	0.163	0.072	0.16	0.078	0.007	0.941	0.072	0.432	−0.031	0.735
Δ_Stress Strain Index (2nd version)	−0.042	0.643	0.082	0.369	−0.079	0.385	0.013	0.886	−0.158	0.082	−0.038	0.675	−0.044	0.631	0.018	0.847
Δ_Stiffness parameter in HC	0.106	0.245	0.079	0.39	0.098	0.284	0.159	0.081	−0.029	0.75	−0.033	0.717	−0.086	0.344	−0.099	0.28
Δ_K1 (D):	0.044	0.628	0.077	0.396	0.023	0.798	0.016	0.86	−0.045	0.621	−0.056	0.533	−0.07	0.443	−0.073	0.419
Δ_K2 (D):	0.191	0.034 *	0.162	0.072	−0.092	0.311	−0.004	0.968	−0.009	0.922	0.071	0.43	−0.108	0.234	−0.082	0.364
Δ_SE	0.078	0.391	0.168	0.062	0.063	0.489	0.028	0.757	0.067	0.461	0.037	0.683	0.137	0.13	0.07	0.437
Δ_AL	−0.053	0.555	−0.019	0.834	−0.013	0.885	−0.035	0.699	0.05	0.576	0.031	0.732	0.051	0.573	0.065	0.473
Δ_VCL	−0.054	0.548	−0.012	0.898	−0.152	1	−0.003	0.972	0.099	0.272	0.074	0.41	0.068	0.452	0.067	0.46
Δ_RATIO VCL/AL	−0.121	0.178	−0.228	0.01 *	−0.095	0.29	0.079	0.379	0.177	0.048	0.124	0.166	−0.045	0.619	0.126	0.159

Footnotes: AL: Axial Length; VCL: Vitreous Cavity Length; Δ: delta of progression; SE: Spherical Equivalent; K1: keratometry in the flat meridian; K2: Keratometry in the steep meridian; Δ: Delta of variation between baseline and the end of follow-up; * *p* ≤ 0.05; ** *p* ≤ 0.01.

**Table 5:** Correlations between biomechanical parameters and biometric parameters.

	Δ_SE				Δ_AL				Δ_VCL				Δ_RATIO_VCL/AL			
	Pearson		Spearman		Pearson		Spearman		Pearson		Spearman		Pearson		Spearman	
	*r*	*p*	*rho*	*p*	*r*	*p*	*rho*	*p*	*r*	*p*	*rho*	*p*	*r*	*p*	*rho*	*p*
Age at baseline	0.103	0.256	0.1	0.27	−0.122	0.066	−0.221	0.08	−0.175	0.122	−0.221	0.137	−0.105	0.244	−0.155	0.142
Δ_cPachy [µm]	−0.069	0.454	−0.018	0.844	−0.014	0.876	−0.015	0.872	−0.012	0.898	0.011	0.907	0.117	0.198	0.037	0.683
Δ_Deformation AmpΔ_ Max [mm]	0.06	0.516	−0.053	0.565	0.155	0.09	0.065	0.482	0.143	0.119	0.058	0.529	0.092	0.315	0.108	0.236
Δ_A1 Time [ms]	−0.027	0.769	0.071	0.439	9.028 × 10^−4^	0.992	−0.016	0.858	0.003	0.978	−0.037	0.687	−0.121	0.185	−0.011	0.901
Δ_A1 Velocity [m/s]	0.151	0.1	0.068	0.462	0.098	0.285	0.078	0.396	0.092	0.317	0.053	0.566	0.112	0.22	0.168	0.064
Δ_A2 Time [ms]	0.008	0.93	−0.046	0.62	0.188	0.041 *	0.155	0.092	0.169	0.066	0.13	0.159	0.06	0.515	0.127	0.166
Δ_A2 Velocity [m/s]	0.01	0.912	0.114	0.221	−0.194	0.035 *	−0.13	0.159	−0.197	0.032 *	−0.163	0.077	−0.068	0.461	−0.097	0.293
Δ_HC Time [ms]	−0.006	0.951	0.011	0.905	−0.025	0.784	−0.032	0.729	−0.037	0.69	−0.019	0.838	−0.133	0.145	−0.031	0.734
Δ_Peak DistΔ_ [mm]	0.038	0.681	−0.048	0.599	0.208	0.022 *	0.148	0.106	0.188	0.039 *	0.122	0.183	0.108	0.238	0.103	0.26
Δ_Radius [mm]	−0.059	0.522	0.022	0.81	0.005	0.956	−0.024	0.797	−0.024	0.79	−0.056	0.543	0.067	0.464	0.121	0.183
Δ_A1 Deformation AmpΔ_ [mm]	0.162	0.076	0.101	0.27	0.023	0.806	−0.084	0.361	0.015	0.874	−0.121	0.185	0.006	0.949	0.156	0.086
Δ_HC Deformation AmpΔ_ [mm]	0.06	0.516	−0.053	0.565	0.155	0.09	0.065	0.482	0.143	0.119	0.058	0.529	0.092	0.315	0.108	0.236
Δ_A2 Deformation AmpΔ_ [mm]	0.103	0.267	0.093	0.315	−0.136	0.142	−0.177	0.054	−0.134	0.147	−0.183	0.047 *	−0.018	0.845	0.068	0.461
Δ_A1 Deflection Length [mm]	−0.018	0.85	−0.073	0.434	0.08	0.387	−0.017	0.851	0.081	0.384	−0.037	0.689	0.009	0.925	0.171	0.062
Δ_HC Deflection Length [mm]	−0.035	0.713	−0.147	0.117	0.144	0.124	0.083	0.377	0.127	0.174	0.067	0.475	0.108	0.246	0.097	0.298
Δ_A2 Deflection Length [mm]	−0.09	0.34	0.004	0.968	−0.093	0.321	−0.113	0.227	−0.043	0.646	−0.067	0.477	0.005	0.955	0.061	0.515
Δ_A1 Deflection AmpΔ_ [mm]	0.059	0.525	−0.022	0.81	0.005	0.956	−0.013	0.888	0.027	0.772	−0.054	0.553	0.008	0.927	0.145	0.111
Δ_HC Deflection AmpΔ_ [mm]	−0.101	0.274	−0.095	0.3	0.081	0.38	0.086	0.351	0.029	0.75	0.058	0.531	0.017	0.856	0.109	0.233
Δ_A2 Deflection AmpΔ_ [mm]	0.028	0.767	0.116	0.211	−0.141	0.125	−0.19	0.039 *	−0.118	0.2	−0.161	0.08	0.002	0.982	0.209	0.022 *
Δ_Deflection AmpΔ_ Max [mm]	−0.103	0.261	−0.13	0.158	0.075	0.411	0.091	0.32	0.025	0.789	0.076	0.409	0.011	0.908	0.088	0.336
Δ_Deflection AmpΔ_ Max [ms]	0.041	0.659	−0.051	0.58	0.111	0.227	0.163	0.074	0.106	0.246	0.161	0.078	−0.001	0.991	0.013	0.886
Δ_Whole Eye Movement Max [mm]	−0.088	0.341	0.003	0.974	0.057	0.534	−0.052	0.571	0.038	0.683	−0.086	0.351	−0.012	0.897	0.02	0.826
Δ_Whole Eye Movement Max [ms]	−0.139	0.13	−0.026	0.776	0.052	0.574	−0.053	0.561	0.049	0.593	−0.055	0.548	0.016	0.862	0.022	0.811
Δ_A1 Deflection Area [mm^2^]	0.037	0.691	0.03	0.748	−0.037	0.69	−0.062	0.498	−0.011	0.905	−0.049	0.595	−0.004	0.967	0.128	0.161
Δ_HC Deflection Area [mm^2^]	0.086	0.35	−0.021	0.821	0.153	0.094	0.071	0.438	0.162	0.076	0.076	0.409	0.081	0.373	0.128	0.16
Δ_A2 Deflection Area [mm^2^]	−0.035	0.708	−0.051	0.587	−0.123	0.184	−0.087	0.349	−0.1	0.279	−0.05	0.592	0.019	0.835	0.224	0.014 *
Δ_A1 dArc Length [mm]	−0.035	0.704	0.013	0.889	−0.019	0.833	0.032	0.731	0.004	0.97	0.093	0.31	−0.006	0.947	−0.179	0.049 *
Δ_HC dArc Length [mm]	−0.048	0.604	−0.1	0.277	−0.102	0.264	0.074	0.422	−0.094	0.306	0.072	0.435	−0.016	0.864	−0.076	0.403
Δ_A2 dArc Length [mm]	−0.047	0.612	−0.058	0.533	−0.124	0.178	−0.056	0.546	−0.117	0.206	−0.055	0.553	−0.015	0.87	−0.197	0.031 *
Δ_dArcLengthMax [mm]	−0.008	0.928	0.007	0.938	−0.014	0.883	0.031	0.738	−0.018	0.842	0.022	0.814	0.019	0.831	−0.043	0.635
Δ_Max InverseRadius [mm^−1^]	−0.169	0.064	−0.101	0.272	−0.058	0.53	0.101	0.272	−0.071	0.44	0.031	0.735	0.01	0.909	0.099	0.277
Δ_DA Ratio Max (2 mm)	−0.097	0.294	−0.083	0.368	0.092	0.317	0.113	0.215	0.082	0.37	0.136	0.138	0.053	0.561	0.011	0.908
Δ_PachySlope [µm]	−0.056	0.54	−0.097	0.293	0.018	0.841	0.023	0.804	0.018	0.847	0.046	0.619	0.03	0.74	0.082	0.37
Δ_DA Ratio Max (1 mm)	−0.144	0.117	−0.089	0.336	−0.149	0.103	−0.021	0.815	−0.13	0.154	0.018	0.842	0.043	0.637	−0.094	0.302
Δ_Ambrosio Relational Thickness	0.155	0.091	0.143	0.119	0.19	0.037 *	0.141	0.123	0.187	0.04 *	0.144	0.115	0.135	0.138	−0.012	0.895
Δ_Biomechanically-corrected IOP	−0.034	0.715	0.067	0.47	−0.018	0.841	−0.041	0.654	−0.016	0.863	−0.067	0.463	−0.112	0.217	−0.013	0.889
Δ_Integrated Radius [mm^−1^]	−0.234	0.01 *	−0.116	0.206	0.156	0.087	0.168	0.065	0.134	0.144	0.134	0.142	0.039	0.666	0.061	0.506
Δ_Stiffness parameter in A1	−0.102	0.271	0.059	0.522	−0.112	0.222	−0.036	0.695	−0.106	0.249	−0.018	0.842	−0.008	0.933	−0.154	0.093
Δ_Corvis biomechanical index	−0.17	0.068	−0.212	0.022 *	−0.101	0.277	−0.064	0.491	−0.07	0.456	−0.031	0.739	−0.148	0.109	−0.034	0.715
Δ_Tomographic and Biomechanical Index	−0.025	0.792	0.003	0.975	−0.144	0.122	−0.166	0.074	−0.1	0.283	−0.139	0.135	−0.013	0.893	0.058	0.532
Δ_Stress Strain Index	0.073	0.432	0.211	0.021 *	−0.256	0.005 **	−0.24	0.008 **	−0.232	0.011 *	−0.187	0.041 *	−0.243	0.007 **	−0.178	0.051
Δ_Biomechanically-corrected IOP (2nd)	0.097	0.291	0.035	0.702	−0.081	0.377	−0.041	0.656	−0.029	0.752	−0.032	0.731	−0.032	0.723	−0.051	0.579
Δ_Stress Strain Index (2nd version)	−0.11	0.233	−0.026	0.778	0.074	0.419	0.009	0.918	0.014	0.88	−0.041	0.656	0.057	0.532	0.132	0.146
Δ_Stiffness parameter in HC	−0.028	0.762	0.103	0.261	−0.072	0.434	−0.056	0.544	−0.052	0.568	−0.041	0.657	−0.084	0.36	−0.041	0.657
Δ_K1 (D):	−0.02	0.826	−0.033	0.72	−0.036	0.689	−0.055	0.545	−0.073	0.423	−0.115	0.207	0.089	0.325	0.035	0.696
Δ_K2 (D):	−0.031	0.733	−0.163	0.073	−0.091	0.315	0.085	0.995	−0.062	0.497	0.003	0.973	−0.008	0.927	−0.142	0.115

Footnotes: AL: Axial Length; VCL: Vitreous Cavity Length; Δ: delta of progression; SE: Spherical Equivalent; K1: keratometry in the flat meridian; K2: Keratometry in the steep meridian; Δ: Delta of variation between baseline and the end of follow-up; * *p* ≤ 0.05; ** *p* ≤ 0.01.

## Data Availability

The data supporting the findings of this study are available from the corresponding author upon reasonable request.

## Supplementary Materials

The following supporting information can be downloaded at: https://www.mdpi.com/article/10.3390/bioengineering13030367/s1, Questionnaire “Oporto Myopia Study” (PDF file).
