# Supplementary material for: Changes in Ocular Biomechanics During Adolescence and Its Relationship with Lifestyle and Myopic Progression: The Oporto Myopia Study
Source: Bioengineering (Basel). 2026 Mar 20;13(3):367. doi: 10.3390/bioengineering13030367 (PMC13023756; doi:10.3390/bioengineering13030367)
Supplement: Supplementary file 1 [file bioengineering-13-00367-s001.zip › QUESTIONNAIRE_ OPORTO MYOPIA STUDY.pdf]

QUESTIONNAIRE: OPORTO MYOPIA STUDY

This questionnaire consists of 33 quick and simple questions (3–5 minutes). Its purpose is statistical use, serving as a tool to continually improve clinical practice and ocular health among patients of the Ophthalmology Department at Centro Hospitalar e Universitário de Santo António (Unidade Local de Saúde de Santo António).

All data will be analyzed anonymously, only for statistical purposes, without identifying individual responses.

The questionnaire is divided into 5 parts:

- 1. GENERAL DEMOGRAPHIC DATA – 8 questions
- 2. GENERAL HEALTH DATA – 2 questions
- 3. OCULAR HEALTH DATA – 6 questions
- 4. LIFESTYLE DURING CHILDHOOD AND ADOLESCENCE – 7 questions
- 5. CURRENT LIFESTYLE DATA – 10 questions

** Indicates a required question*

PART 1 — GENERAL DEMOGRAPHIC DATA

1. INITIALS OF NAME *

---

2. CLINICAL RECORD NUMBER *

---

3. COUNTRY OF BIRTH

---

4. GENDER *

- ■ Female
- ■ Male
- ■ Unspecified

5. AGE *

---

6. HEIGHT (centimeters) *

---

7. WEIGHT (kilograms) *

---

8. MUNICIPALITY OF RESIDENCE *

---

9. POSTAL CODE (first 4 digits) *

---

10. ACADEMIC DEGREE

- ■ 4th grade
- ■ 6th grade
- ■ 9th grade
- ■ 12th grade
- ■ Bachelor’s / Master’s
- ■ Doctorate

PART 2 — GENERAL HEALTH DATA

11. HISTORY OF ALLERGIC DISEASES (check all that apply) *

- ■ None
- ■ Atopy
- ■ Rhinitis
- ■ Asthma
- ■ Dermatitis
- ■ Other: _______

12. HISTORY OF AUTOIMMUNE OR CONNECTIVE TISSUE DISEASES (check all that apply) *

- ■ None
- ■ Rheumatoid arthritis
- ■ Lupus
- ■ Spondylitis
- ■ Thyroid disease
- ■ Polymyositis / Dermatomyositis
- ■ Marfan syndrome
- ■ Pseudoxanthoma elasticum
- ■ Ehlers-Danlos syndrome
- ■ Other: _______

PART 3 — OCULAR HEALTH DATA

13. DO YOU USUALLY HAVE EYE SYMPTOMS IN THE RIGHT EYE? (check all that apply) *

- ■ No symptoms
- ■ Itching (pruritus)
- ■ Stinging
- ■ Grittiness
- ■ Dry eye sensation
- ■ Blurred vision sensation
- ■ Tearing
- ■ Red eye
- ■ Discharge
- ■ Photophobia (marked light discomfort)
- ■ Blurred vision at certain times of day that improves after blinking
- ■ Other: _______

14. DO YOU USUALLY HAVE EYE SYMPTOMS IN THE LEFT EYE? (check all that apply) *

- ■ No symptoms
- ■ Itching (pruritus)
- ■ Stinging
- ■ Grittiness
- ■ Dry eye sensation
- ■ Blurred vision sensation
- ■ Tearing
- ■ Red eye
- ■ Discharge
- ■ Photophobia (marked light discomfort)
- ■ Blurred vision at certain times of day that improves after blinking
- ■ Other: _______

15. DO YOU USE ANY EYE DROPS IN THE RIGHT EYE? *

- ■ Yes
- ■ No

If yes, please specify: _______

16. DO YOU USE ANY EYE DROPS IN THE LEFT EYE? *

- ■ Yes
- ■ No

If yes, please specify: _______

17. FAMILY HISTORY OF MYOPIA

- ■ Yes
- ■ No
- ■ Don’t know

18. PERSONAL HISTORY OF CORNEAL ECTASIA (e.g., keratoconus) *

- ■ No
- ■ Yes, right eye
- ■ Yes, left eye

■ Yes, both eyes

19. FAMILY HISTORY OF CORNEAL ECTASIA (e.g., keratoconus) *

■ Yes
■ No
■ Don’t know

20. USE OF CONTACT LENSES *

■ No
■ Right eye
■ Left eye
■ Both eyes

PART 4 — LIFESTYLE DURING CHILDHOOD AND ADOLESCENCE

21. TYPE OF SCHOOL ATTENDED MOSTLY DURING CHILDHOOD/ADOLESCENCE

- ■ Public
- ■ Private

22. WHERE DID YOU USUALLY SPEND YOUR RECESS TIME?

- ■ Indoors
- ■ Outdoors

23. AGE WHEN YOU STARTED USING TOUCHSCREEN DEVICES (PHONE/TABLET)

- ■ Up to 5 years
- ■ 5–9 years
- ■ 10–14 years
- ■ 15–20 years
- ■ After 20 years

24. HABITUAL USE OF VIDEO GAME CONSOLES DURING CHILDHOOD/ADOLESCENCE

- ■ Yes
- ■ No

25. DID YOU REGULARLY PRACTICE OUTDOOR EXTRACURRICULAR ACTIVITIES DURING CHILDHOOD/ADOLESCENCE (sports, recreational activities, outdoor play…)?

- ■ Yes
- ■ No

26. DID YOU REGULARLY PRACTICE NEAR-VISION EXTRACURRICULAR ACTIVITIES DURING CHILDHOOD/ADOLESCENCE (language learning, reading/studying, musical instruments, drawing/painting, other arts…)?

- ■ Yes
- ■ No

27. WHICH TYPE OF EXTRACURRICULAR ACTIVITIES WAS MOST PREDOMINANT DURING CHILDHOOD/ADOLESCENCE?

- ■ Outdoor activities
- ■ Near-vision activities

PART 5 — CURRENT LIFESTYLE DATA

28. ON AVERAGE, HOW MANY HOURS PER WEEK DO YOU SPEND OUTDOORS? *

- ■ Never
- ■ 1–5 hours
- ■ 5–10 hours
- ■ 10–15 hours
- ■ 15–20 hours
- ■ 20–25 hours
- ■ 25–30 hours
- ■ More than 30 hours

29. WHEN SPENDING TIME OUTDOORS, DO YOU WEAR SUNGLASSES? *

- ■ Never
- ■ Less than half of the time
- ■ More than half of the time

30. ON AVERAGE, HOW MANY HOURS PER WEEK DO YOU PRACTICE PHYSICAL ACTIVITY? *

- ■ Never
- ■ 1–2 hours
- ■ 3–4 hours
- ■ 5–6 hours
- ■ 7–8 hours
- ■ 9–10 hours
- ■ More than 10 hours

31. ARE YOU REGULARLY EXPOSED TO AIR CONDITIONING AT HOME AND/OR AT WORK? *

- ■ Yes
- ■ No

32. DOES YOUR JOB INCLUDE THE USE OF A COMPUTER OR OTHER SCREENS? *

- ■ Yes
- ■ No

33. ON AVERAGE, HOW MANY HOURS PER DAY DO YOU USE SCREENS? *

- ■ Never
- ■ 1–2 hours
- ■ 3–4 hours
- ■ 5–6 hours
- ■ 7–8 hours
- ■ 9–10 hours
- ■ More than 10 hours

34. ON AVERAGE, HOW MANY HOURS PER DAY DO YOU SPEND READING OR WRITING? *

- ■ Never
- ■ 1–2 hours

- 3–4 hours
- 5–6 hours
- 7–8 hours
- 9–10 hours
- More than 10 hours

35. WHEN SLEEPING, WHICH POSITION IS MOST COMMON? *

- Face down
- Face up
- No predominance

36. ON MOST DAYS, WHICH SIDE OF YOUR FACE IS ON THE PILLOW? *

- Right side
- Left side
- No predominance

37. HOW OFTEN DO YOU RUB/SCRATCH YOUR RIGHT EYE? *

- No
- 1–5 times/day
- 6–10 times/day
- 11–15 times/day
- 16–20 times/day
- More than 20 times/day

38. HOW OFTEN DO YOU RUB/SCRATCH YOUR LEFT EYE? *

- No
- 1–5 times/day
- 6–10 times/day
- 11–15 times/day
- 16–20 times/day
- More than 20 times/day

This content was not created or approved by Google.
